# Comprehensive study on the differential extraction and comparison of bioactive health potential of the Broccoli (*Brassica oleracea*)

**DOI:** 10.7150/ijms.92456

**Published:** 2024-01-21

**Authors:** Durga Uvaraj, Naiyf S. Alharbi, Shine Kadaikunnan, Muthu Thiruvengadam, Baskar Venkidasamy

**Affiliations:** 1 Saveetha Medical College and Hospitals, Saveetha Institute of Medical and Technical Sciences (SIMATS), Saveetha University, Chennai, 600077, India. D.U.; 2 Department of Botany and Microbiology, College of Science, King Saud University, P. O. Box 2455, Riyadh 11451, Saudi Arabia.; 3 Department of Crop Science, College of Sanghuh Life Science, Konkuk University, Seoul, Republic of Korea.; 4 Department of Oral and Maxillofacial Surgery, Saveetha Dental College and Hospitals, Saveetha Institute of Medical and Technical Sciences (SIMATS), Saveetha University, Chennai, 600077, India.

**Keywords:** broccoli, phenolic, flavonoid, antioxidants, anti-inflammatory, antimicrobial, sustainable.

## Abstract

**Introduction:** Broccoli is a cruciferous vegetable that has been shown to have numerous potential therapeutic benefits because of its bioactive compounds.

**Methods:** In this study, we compared the bioactive efficacy of cooked and uncooked (fresh) stems and florets of broccoli extracted with three different solvents: acetonitrile, methanol, and aqueous extracts. The extraction yield and antioxidant and antibacterial potential of different broccoli extracts were examined.

**Results:** Fresh and boiled floret stem extracts increased the extraction yield. The extraction yields were higher for the methanol and acetonitrile extracts than for the aqueous extracts. The antioxidant efficacy of the different extracts was studied using ABTS, DPPH, and metal ion reduction assays. The acetonitrile and aqueous extracts exhibited higher antioxidant activities than the methanolic extracts in different antioxidant assays. In addition, increased antioxidant activity was observed in fresh florets and boiled broccoli stems. TPC and TFC contents were higher in the methanolic extracts than in the aqueous extracts. Similar to antioxidant activities, anti-inflammatory activities were found to be higher in the acetonitrile and aqueous extracts, particularly in boiled stems and fresh florets. Broccoli extracts have been shown to be active against *Bacillus subtilis* and moderately effective against *Pseudomonas aeruginosa* and *Staphylococcus aureus*.

**Conclusions:** Acetonitrile and aqueous extraction of broccoli might be an ideal choice for extraction methods, which show increased extraction yield and antioxidant and anti-inflammatory potentials. Utilization of phytomolecules from natural sources is a promising alternative approach to synthetic drug development.

## Introduction

Brassicaceae, often called the cruciferea or mustard family, is an economically crucial family with 372 genera and 4060 species 1. It possesses various plants like, bok choy (*Brassica rapa*, var. *chinensis*), Brussels sprouts *(B. oleracea,* var. *gemmifera*), bittercress (*Cardamine sp*.), brown mustard (*B. juncea*), broccoli (*B. oleracea*, var. *italica*), cabbage (*B. oleracea*, var. *capitata*), etc., 2. Some of the major varieties of *Brassica oleracea* are broccoli, Brussels sprouts, cabbage, cauliflower, kale, and kohlrabi 3. One of the most assured Indian cultivars grown in commercial cash crops in India is Brassica olarecea 4. Broccoli is considered an important vegetable in the market because of its high nutritional value and elevated levels of phytochemicals, such as glucosinolates and isothiocyanates, which have significant effects on preventing several cancer types and other infections 5. It is also rich in nutrients, including provitamin A (beta carotene), vitamin C (ascorbate), and vitamin E (tocopherol) 6. The ingestion of these vegetables known to decrease risk of specific diseases due to presence of these beneficial biological molecules like vitamins, carotenoids, polyphenols, glucosinolates, flavonoids, and enzymes like peroxidase, cystinelyases, leases etc., 7. These compounds may vary not only based on the crop cycle but also depend on harvest practices, post-harvest storage conditions, or food preparation methods 8.

Many studies have correlated its enhanced bioactivity with properties related to broccoli, such as anticancer, antioxidant, antimicrobial, anti-inflammatory, and antihypertensive activities 9. Additionally, broccoli exhibits anti-obesity, anticarcinogenic, antidiabetic, hepatoprotective, cardioprotective, gastroprotective, antiamnesic, and immunomodulatory properties. The presence of these beneficial components also helps in the treatment of other health-related issues, such as hypercholesterolemia, cardiovascular disease, diabetes, and photosensitivity disorders 10. In the realm of cardiovascular health, broccoli extracts have shown promise for reducing oxidative stress and inflammation. Glucoraphanin, another key compound, is converted to sulforaphane upon consumption, which contributes to its antioxidant properties. This conversion also triggers the stimulation of NRF2, a master regulator of antioxidant responses 11. The stem and inflorescence of broccoli have a higher suppressive impact on pathogenic bacteria, such as *Listeria innocua, Bacillus cereus*, and *Staphylococcus aureus* 12. A recent study reported that broccoli stems contain ascorbic acid, malic acid, phenolic compounds, and caffeic acid, which exhibit inhibitory effects against *L. monocytogenes* 13. Moreover, sulforaphane, found in broccoli, has a potent bacteriostatic effect against *Helicobacter pylori*, an important causative agent of gastric ulcers. Additionally, previous studies have shown that broccoli extracts exhibit anti-inflammatory activity in lipopolysaccharide-stimulated RAW264.7 cells, as evidenced by a reduction in the production of pro-inflammatory cytokines, including IL-6 and TNF-α 14.

Extraction methods and appropriate solvents are essential to achieve the optimal yield and specificity of the desired bioactive constituents from plants 15. Aqueous and methanolic extracts are widely utilized in plant extraction analyses. Extracts that are rich in flavonoids, terpenoids, and phenolic compounds have shown promising anti-inflammatory properties. These compounds modulate inflammatory pathways by inhibiting key enzymes and cytokines, which has been attributed to their potential for mitigating chronic inflammatory diseases 16. Plant extracts containing essential oils, alkaloids, and tannins have demonstrated potent antimicrobial effects 17. These extracts can inhibit the growth of bacteria and even drug-resistant strains, thereby offering an alternative approach to combat microbial infections. Phytochemicals such as polyphenols and carotenoids contribute to the antioxidant activity of plant extracts. They neutralize reactive oxygen species (ROS), protect cells from oxidative damage, and potentially decrease the risk of chronic diseases associated with oxidative stress 18.

The main objective of this study was to compare the extraction yield and antioxidant, anti-inflammatory, and antimicrobial properties of fresh and cooked broccoli stems and florets extracted with different solvents. Various antioxidant assays were performed to determine antioxidant potential, and a protein denaturation inhibition assay was performed to evaluate anti-inflammatory activity. The antimicrobial performance of the extracts was examined by live and dead bacterial analyses.

## Materials and Methodology

### Collection and extraction of broccoli samples

Broccoli (*Brassica oleracea*) samples were purchased from a supermarket in Coimbatore, India. The broccoli samples were stored in a cool, dry place prior to analysis. Broccoli samples were washed thoroughly with sterile distilled water. The stems and florets of broccoli were separated. The cooked (boiled at 100°C for 10 min) and uncooked (fresh) broccoli stems and florets were ground with liquid nitrogen, and fine powdered samples (1 g) were extracted with 10 mL of three different solvents: methanol (70%), acetonitrile, and aqueous (sterile water). The extraction yields were examined at different time intervals (0, 12, and 24 h) to determine the optimal extraction yield. For various biochemical analyses, broccoli samples were extracted for 12 h with different solvents in an orbital shaker at 120 rpm, followed by centrifugation at 2000 rpm for 5 min, and the supernatants were collected and stored at 4°C. The collected supernatants were concentrated in a rotary evaporator (Rotavac evaporator, Eppendorf Germany) and dissolved in methanol at the concentrations of 0.1 g/mL for different biochemical analysis.

### Determination of total phenolic content

The total phenolic content (TPC) was determined using the Folin-ciocalteu method 19. The extracted broccoli samples (0.5 mL) were mixed with 1 mL of 0.5M sodium bicarbonate and incubated for 2 h at RT, and the absorbance was evaluated at 765 nm. Total phenolic content was calculated using the gallic acid standard. The phenolic content was expressed as mg/g gallic acid equivalent to the fresh weight (mg/g GAE FW).

### Determination of total flavonoid content

Total flavonoid content (TFC) was analyzed by the flavonoid-aluminium complex formation method 20. Broccoli extracts (0.5 mL) were mixed with 2.25 mL distilled water, 0.25 mL of 5% aluminium chloride and 1 mL 1M NaOH. The absorbance of the mixture was evaluated at a wavelength of 510 nm. TFC was measured using quercetin as a standard and expressed as mg/g quercetin equivalent to fresh weight (mg/g QE FW).

### Antioxidant assays

#### DPPH assay

The antioxidant activities of the different extracts of the stem and floret parts of broccoli were calculated using the DPPH assay. DPPH scavenging activity was measured using the method described by Gudiño et al. 21. The sample extract (0.5 mL) and DPPH-methanolic solution (0.5 ml) were mixed and stored in the dark for 30 min, followed by spectrophotometric measurements at 517 nm. A mixture of 500 µL of methanol and 500 µL of DPPH solution served as a control. The DPPH radical scavenging ability of the samples was calculated as (DSE %) = [(A0-A1)/A0×100], where A0 is the absorbance of the control and A1 is the absorbance in the presence of the sample.

#### ABTS radical scavenging assay

The ABTS radical scavenging assay involves the use of ABTS (2,2'-azino-bis(3-ethylbenzothiazoline-6-sulphonic acid)) as the free radical, which is reduced by an antioxidant. ABTS assay was performed as described by Re et al. 22. ABTS solution was prepared by combining 2.45 mM potassium persulfate (K_2_S_2_O_8_) with 7 mM ABTS in deionized water. The combination was allowed to react overnight in the dark to generate the ABTS radical cation. The broccoli extracts (20 µL) were added to 2 mL of ABTS radical solution and incubated at RT for six mins followed by the absorbance was recorded at 734 nm. The control included 20 µL methanol and 2 mL ABTS radical solution.

The formula (DSE%) = [(A0-A1)/A0] × 100 was used to determine the percentage of ABTS radical scavenging activity, where A0 represents the absorbance of the control and A1 represents the absorbance in the presence of the sample. The proportion of ABTS radical inhibition represents antioxidant activity.

#### Metal ion reduction assay

Nile et al. 23 carried out a metal ion reduction assay in broccoli. Broccoli extracts (100 µL) were added to 0.5% v/v DMSO and 5 ml of a potassium ferricyanide (1 mM) solution and kept at RT for 30 min. The reaction was stopped by the addition of 3 mL of 10% TCA. Further, 5 mL of distilled water and 1 mL of FeCl_3_ solution (0.01%) were added to the reaction mixture and kept at RT for 10 min, followed by measurement of the absorbance at 593 nm using the appropriate blank solution. Trolox (100-2000 mM) was used to create the calibration curve, with the outcomes represented in 1 mmol Trolox/g extract.

### Measurements of antibacterial activity

An antibacterial study was carried out using the approach of Chen et al. 24 to discriminate between dead and live bacterial cells. The antimicrobial ability of different broccoli extracts was investigated against human pathogens, such as gram-positive bacteria (*Bacillus cereus* and *Staphylococcus aureus*) and gram-negative bacteria (*Pseudomonas aeruginosa* and *Escherichia coli*). Approximately 100 µL of bacterial cell suspensions (1.5 x 10^8^ CFU mL^-1^) were subjected to different extracts of broccoli at 100 µL concentrations and stored at 37± 2°C for 24 h. It was then spun at 5000 rpm at 4°C for 5 min, and the pellet was cleaned three times using sterile PBS. The samples were then combined with a 1:1 solution of ethidium bromide (EB) and acridine orange (AO), and the reaction was allowed to proceed for 30 min. The cell suspension (5 µL) was encased between a glass slide and cover slip, washed with PBS, and examined under a fluorescence microscope at 100X magnification with an excitation filter ranging from 430 to 470 nm.

### Statistical analysis

The values are presented as means of triplicate analyses of the samples (n = 3) plus standard deviation. Using SPSS, a statistical package application, the data were further investigated using one-way analysis of variance (ANOVA) followed by Duncan's multiple range test (P<0.05). Significant differences between the varieties or in comparison with the control were determined using P < 0.05.

## Results

### Extraction yield

**Figure 1:**
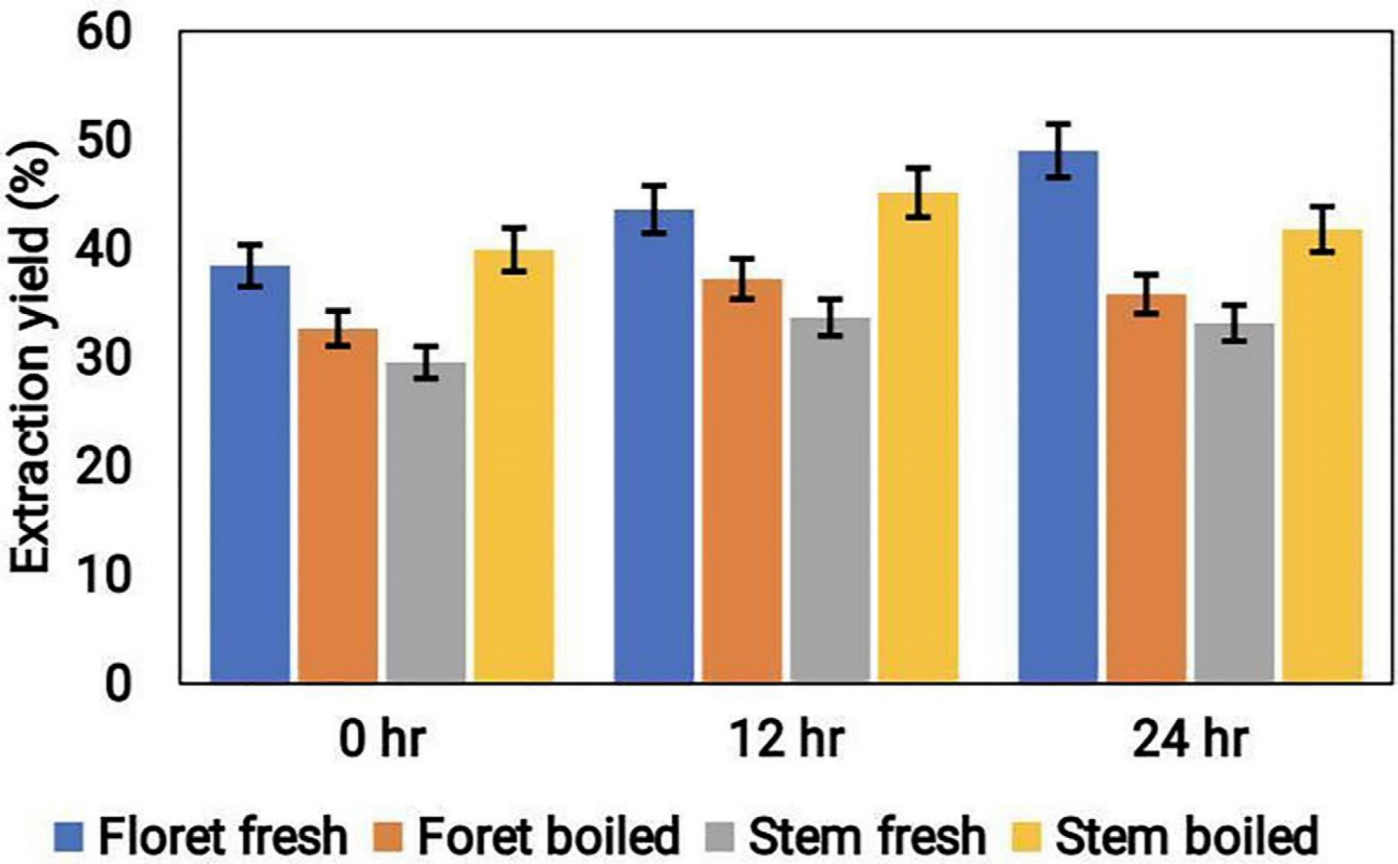
The extraction yield percentage of cooked (boiled floret and stems) and uncooked (fresh floret and stems) broccoli samples at different time intervals of duration (hrs).

The extraction yield of broccoli samples in three different solvents (aqueous, acetonitrile, and methanol) was assessed at different time intervals. The results showed that methanol extraction showed the highest yield of 33.36 ± 0.86%, followed by acetonitrile with 30.21 ± 0.70%, and the aqueous extraction with a yield of 24.84 ± 0.57% (Fig. 1). There was no significant variation in extraction yield after 12 and 24 h of incubation. Thus, all other biochemical analyses were performed on samples extracted for 12 h. These results indicate that the polarity of the solvent plays a vital role in the extraction yield of broccoli.

### Evaluation of antioxidant properties

#### DPPH radical scavenging assay

**Figure 2:**
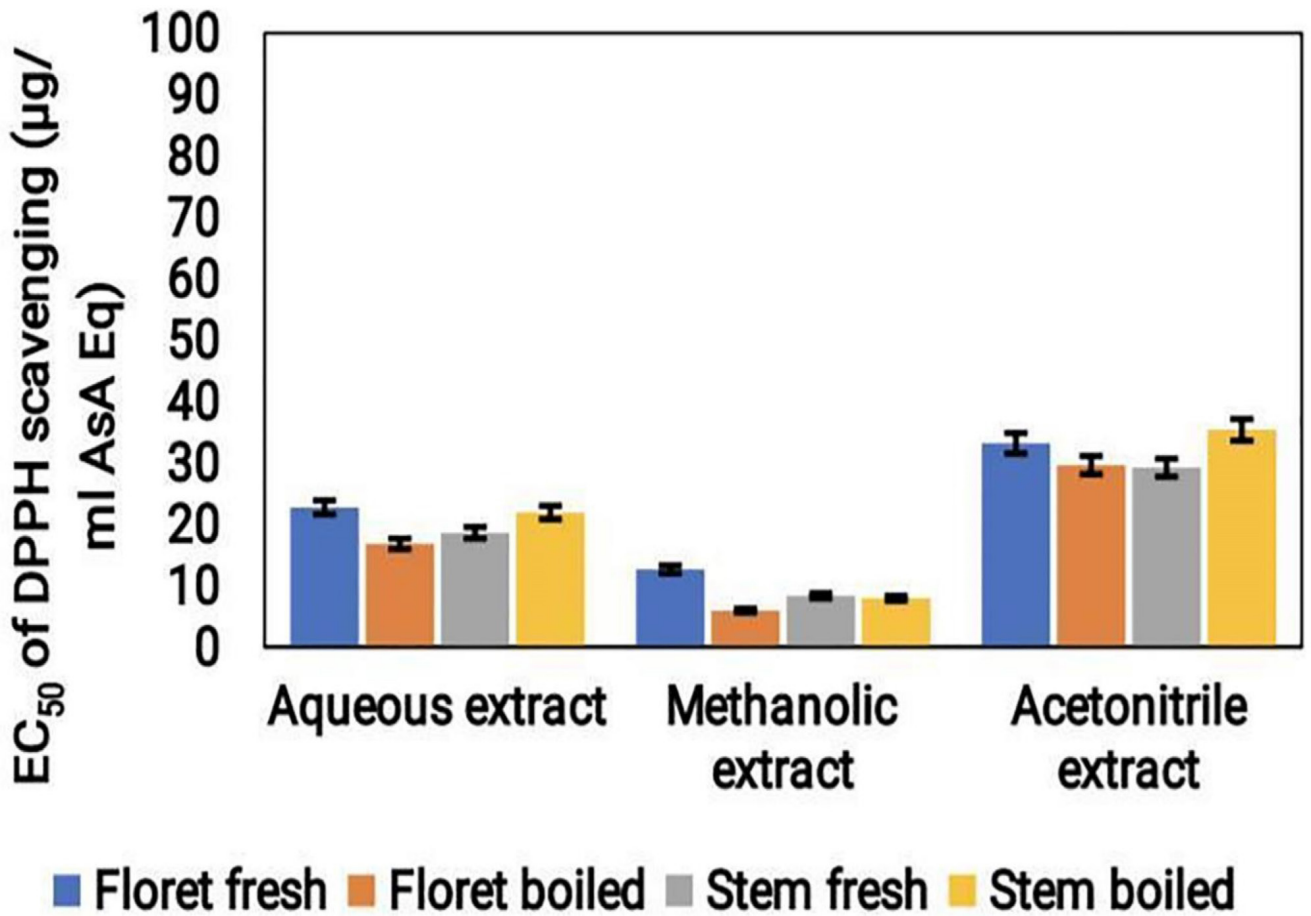
DPPH radical scavenging activity (μg/mL ASA Eq) of cooked (boiled floret and stems) and uncooked (fresh floret and stems) broccoli samples extracted with various solvents.

The antioxidant activity of broccoli samples was assessed using the DPPH assay. The outcomes revealed that the highest antioxidant property was observed in the acetonitrile extract with a value of 40 μg/mL ASA Eq. The aqueous extract showed moderate antioxidant activity (20 μg/mL ASA Eq), while the methanolic extract demonstrated the least amount of antioxidant activity (10 μg/mL ASA Eq) (Fig. 2). Moreover, fresh florets and boiled stems exhibited increased DPPH scavenging activity.

#### ABTS radical scavenging assay

**Figure 3:**
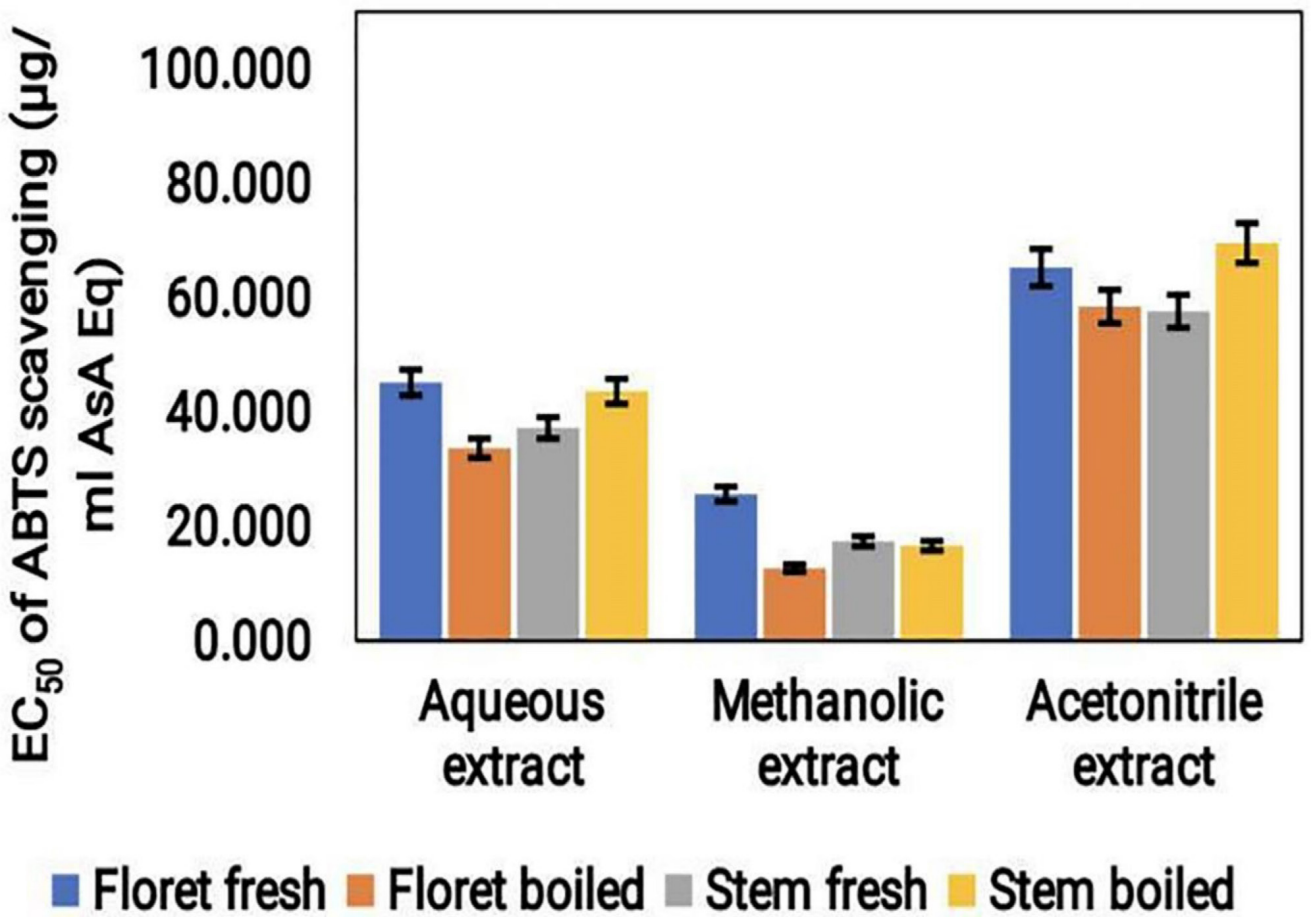
Graphical representation of ABTS radical scavenging activity (μg/mL ASA Eq) of cooked (boiled floret and stems) and uncooked (fresh floret and stems) broccoli samples extracted with different solvents.

According to ABTS assay results, the acetonitrile extract of the broccoli sample had the highest antioxidant activity, with a value of approximately 70 μg/mL ASA Eq. This was followed by the aqueous extract with a value of approximately 40 μg/mL ASA Eq, and the methanol extract with a value of approximately 30 μg/mL ASA Eq (Fig. 3). Notably, the antioxidant capacity of the methanol extract was the lowest of the three tested extracts. Furthermore, fresh florets and boiled stem showed higher ABTS scavenging activities. These findings imply that, similar to the DPPH assay, ABTS scavenging results also exhibited higher antioxidant activity in acetonitrile extracts.

#### Metal ion reduction assay

**Figure 4:**
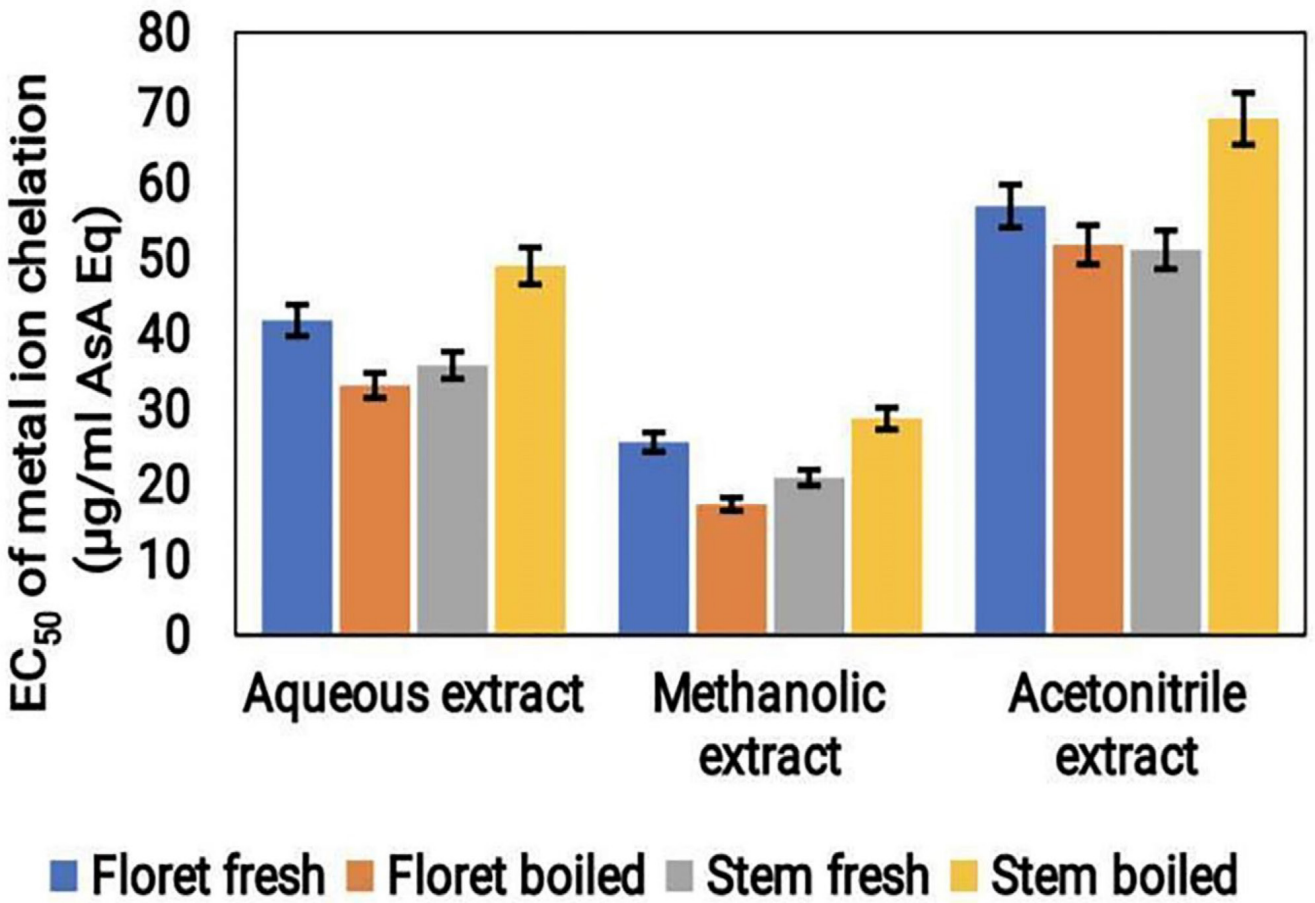
Metal ion chelation activity (μg/mL ASA Eq) of the cooked (boiled floret and stems) and uncooked (fresh floret and stems) broccoli samples extracted using various solvents

The metal ion reduction potential of broccoli samples under both cooked and uncooked conditions was recorded using an EC50 assay. Among the three extracts tested, the acetonitrile extract showed the highest metal ion reduction potential, with an EC50 value of approximately 70 μg/mL ASA Eq. This was followed by the aqueous extract, which had an EC50 value of around 50 μg/mL ASA Eq. The methanol extract showed the lowest metal ion reduction potential with an EC50 value of approximately 30 μg/mL ASA Eq (Fig.4). These findings indicate that acetonitrile may be a more effective solvent for extracting metal ion reducing compounds from broccoli which showed better results than the aqueous and methanol solvents.

### Determination of total phenolic content

**Figure 5:**
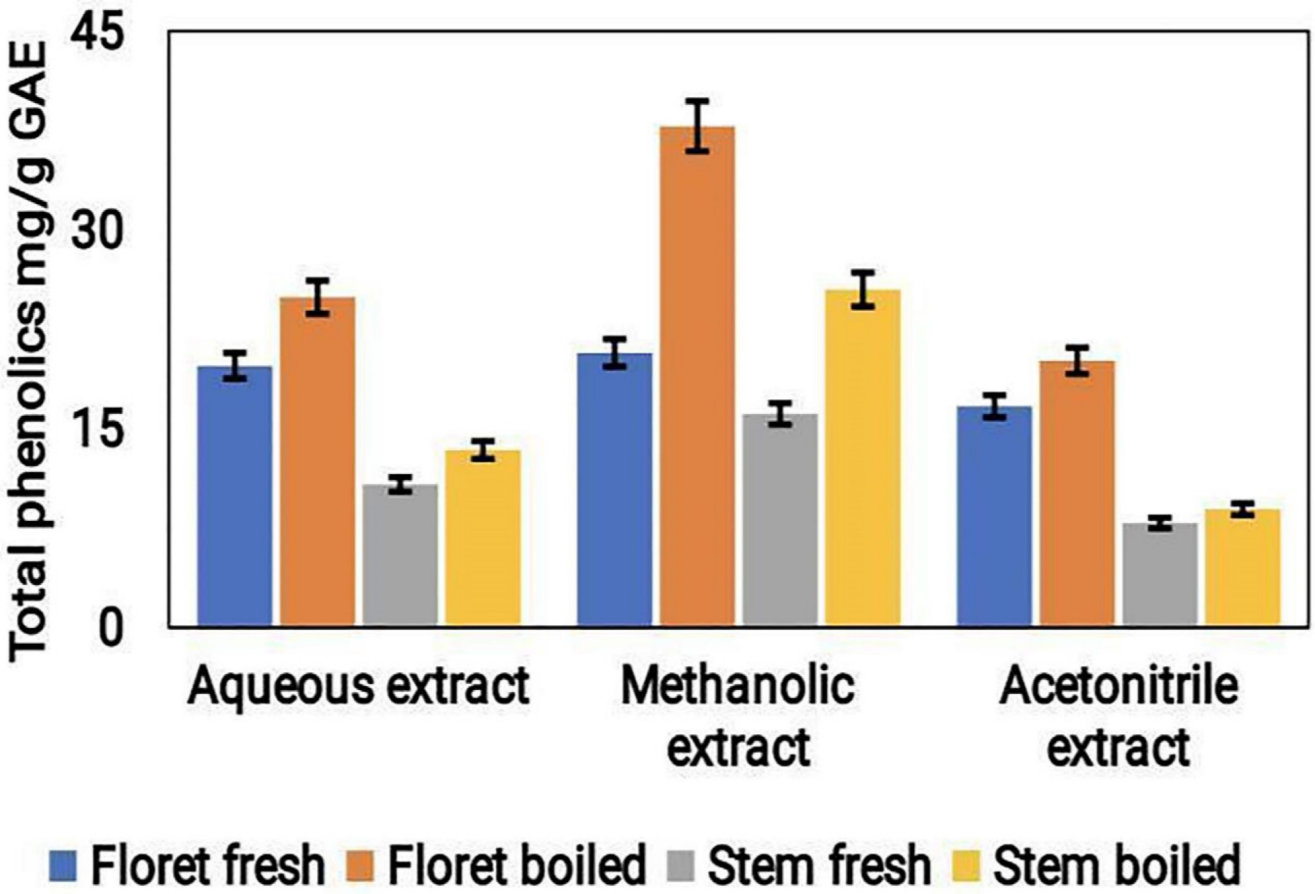
The total phenolic content in the aqueous, acetonitrile, and methanolic extracts of cooked (boiled floret and stems) and uncooked (fresh floret and stems) broccoli samples was expressed as mg/g GAE FW.

The Folin-Ciocalteu method was used to calculate the total phenolic content (TPC) of the broccoli extracts. The findings revealed that the methanolic extract had the highest TPC levels with a value of approximately 0.42 mg/g GAE, followed by the acetonitrile extract with a value of approximately 0.39 mg/g GAE, and the least TPC was found in the aqueous extract with a value of approximately 0.30 mg/g GAE (Fig. 5). The higher TPC values observed in the methanolic and acetonitrile extracts could be attributable to the greater solubility of the phenolic compounds in organic solvents than in water. The results suggest that the choice of extraction solvent significantly affects the phenolic content of broccoli samples under both conditions.

### Analysis of total flavonoid content

**Figure 6:**
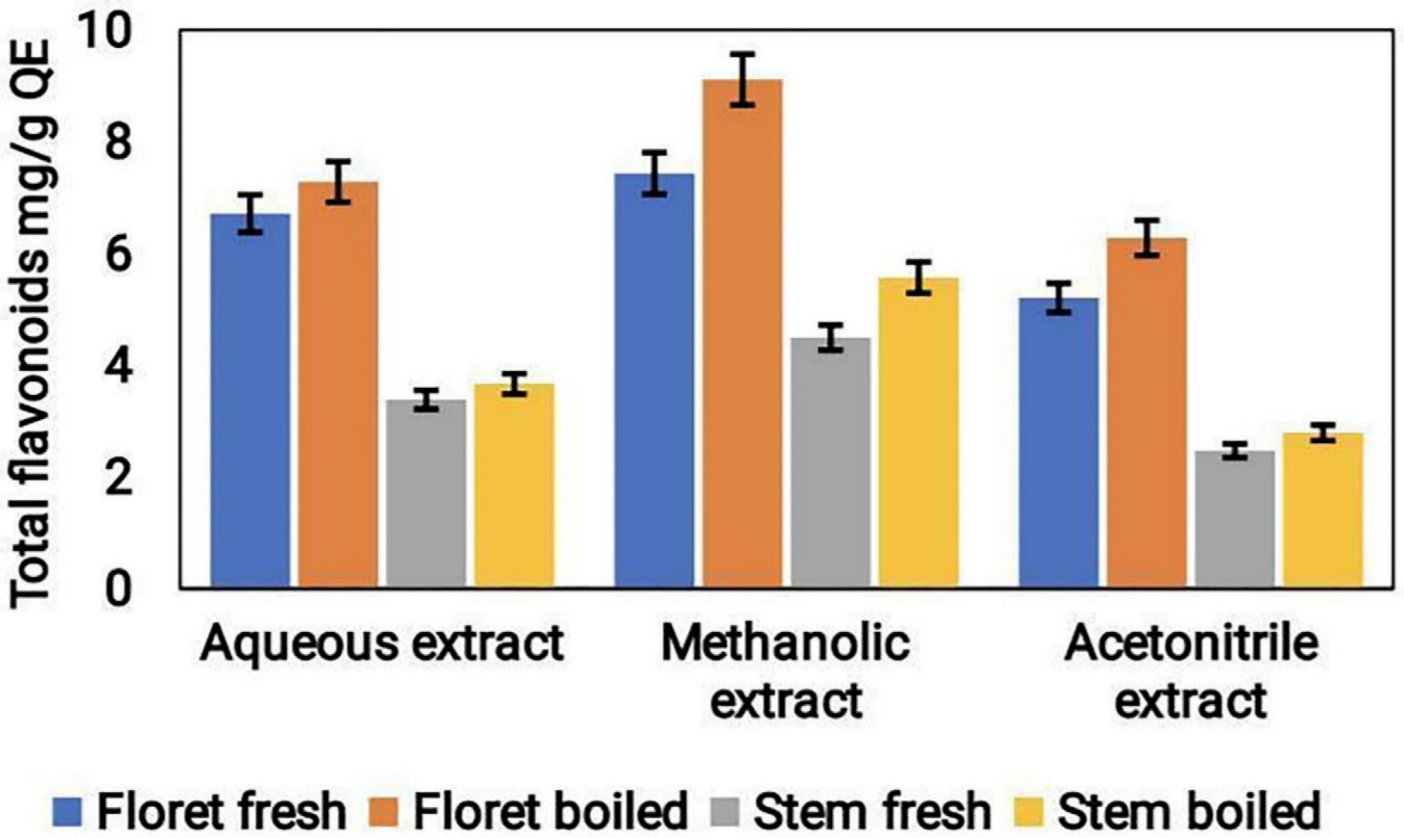
Total flavonoid content in the aqueous, acetonitrile & methanolic extracts of cooked (boiled floret and stems) and uncooked (fresh floret and stems) broccoli samples was expressed as mg/g QE FW.

The total flavonoid content (TFC) of the three various extracts of broccoli samples, likely aqueous, methanol, and acetonitrile, was determined. The outcomes revealed that the TFC was the highest in the methanolic extract followed by the acetonitrile and aqueous extracts. The TFC values were found to be 0.16 mg/g QE, 0.14 mg/g QE, and 0.11 mg/g QE for methanolic, acetonitrile, and aqueous extracts, respectively (Fig. 6). The higher TFC content of the methanolic and acetonitrile extracts can be attributed to the fact that these solvents have a better ability to extract flavonoids from the broccoli samples compared to water.

### Anti-inflammatory properties of broccoli samples

**Figure 7:**
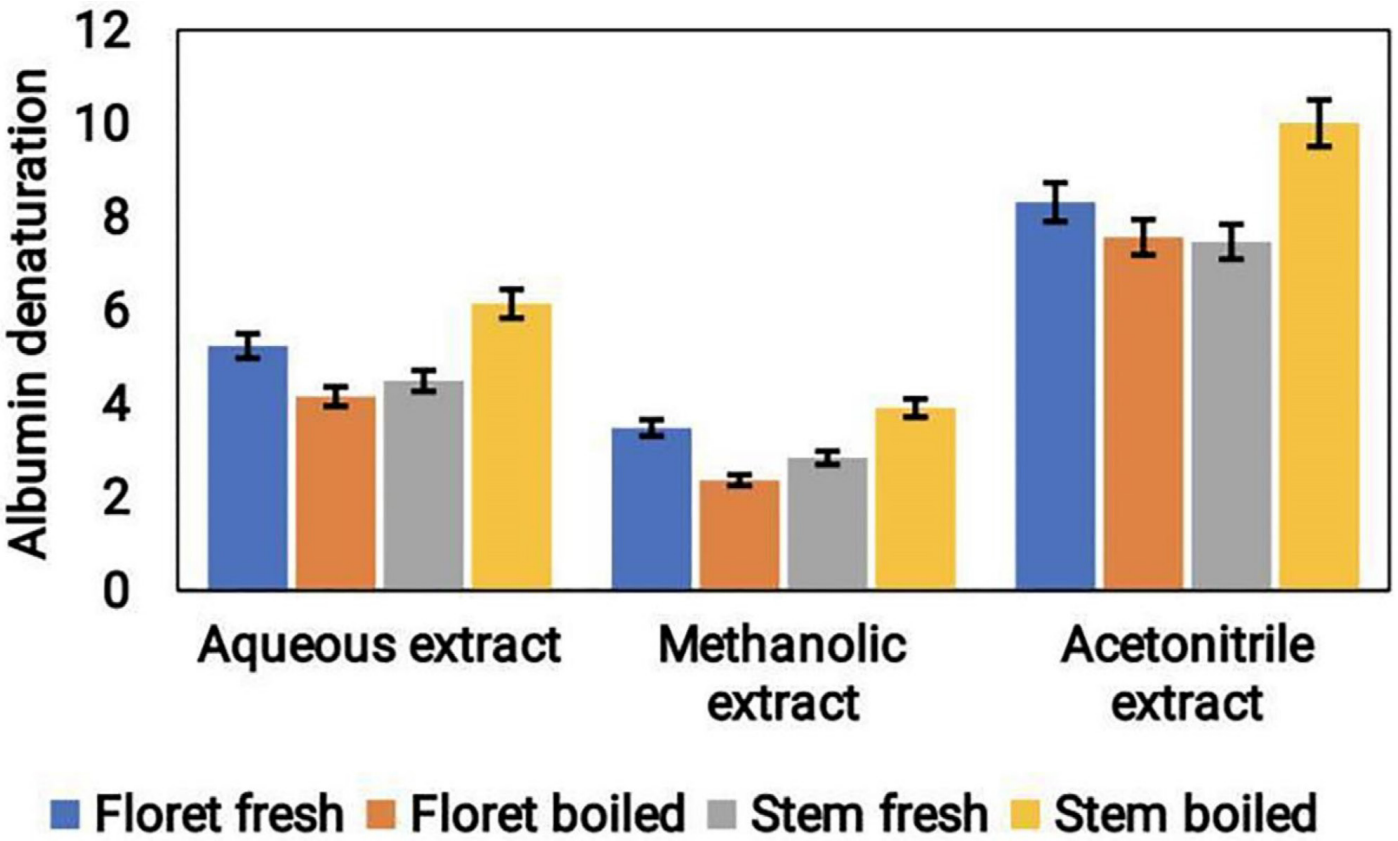
Anti-inflammatory activity of cooked and uncooked broccoli samples extracted using different solvents like aqueous, methanolic and acetonitrile.

The anti-inflammatory potential of different extracts of broccoli was evaluated using the albumin denaturation assay. The results indicated that acetonitrile extract showed the highest level anti-inflammatory action, preceded by aqueous and methanol extracts. The anti-inflammatory potential of the acetonitrile extract was approximately 10 μg/mL, whereas the aqueous and methanol extracts showed values of around 6 μg/mL and 4 μg/mL, respectively (Fig. 7). These findings suggest that broccoli samples have anti-inflammatory potential and that the extraction method plays a significant part in determining the efficacy of the extract. Acetonitrile extract showed the most potent anti-inflammatory activity, indicating that acetonitrile might be an ideal solvent for the extraction of phytocompounds from broccoli samples.

### Evaluation of antimicrobial properties

The antibacterial potential of the broccoli samples extracted using different solvents was examined against clinically important gram positive (*B. subtilis* and* S. aureus*) and gram negative (*K. pneumoniae* and* P. aeruginosa*) bacterial pathogens. The results showed that broccoli samples rapidly more effective against gram-positive than gram-negative pathogens. The figure S1 clearly indicated that there were a higher number of dead cells in all the different types of broccoli samples, in contrast to the control. Broccoli extracts substantially suppressed the growth of *B. subtilis* followed by that of *S. aureus* and *P. aeruginosa*. Interestingly, broccoli extracts were more potent against gram positive pathogens than gram negative bacterial species.

## Discussion

Broccoli by-products have been shown to contain diverse phytoconstituents namely, flavonoids, phenolics, carotenoids and sterols etc., 25, 26. Studies have shown that broccoli extracts possess various health-promoting properties, including antioxidant, antibacterial, and anti-inflammatory activities 14. Extraction is the primary step to remove the required phytomolecules and extraction with solvents is often used for extraction 15. Solvent selection is crucial for extraction and ethanol and methanol are the commonly used solvents for phytochemical extractions. Numerous studies have investigated the antioxidant potential of different extracts of *B. oleracea*. The extraction type, duration, temperature, solvents and concentration can profoundly affect TPC yield 27. A study by Wang et al., 28 reported that the methanolic extract of broccoli had the increased TPC and antioxidant activity compared to the ethyl acetate and water extracts. The methanolic extract (80%) of broccoli showed highest TPC content 29. Similarly, our results also displayed that TPC content was higher in the methanol (70%) and acetonitrile extracts than in the aqueous extracts.

Steaming, microwave and boiling are often used for cooking broccoli, which greatly influences its phytochemical composition. TFC was increased in the microwave treated broccoli florets than in the steamed treatments 30. While the TPC increased in both the microwave and steam treatments. In our study, we observed that the fresh florets exhibited higher antioxidants, TPC and TFC than cooked florets. However, boiled stem showed higher antioxidant, TFC, and TPC than uncooked stems which is consistent with a previous study by López-Hernández et al., 31. The enhanced carotenoid and polyphenol contents were noticed upon boiling in fresh broccoli and Brussels sprouts respectively 32.

The antioxidant potential is highly variable in florets and stems 33. These results indicate that the bioactive properties may vary depending on the type of sample and process used for the study. The results of the DPPH and ABTS assays indicated strong antioxidant potential in the methanolic and acetone extracts of broccoli compared to their aqueous extracts 34. Similarly, in our study, the acetonitrile extract was found to have an increased antioxidant status than the aqueous and methanolic extracts as demonstrated by different antioxidant assays like ABTS, DPPH and metal ion reduction scavenging assays.

Several studies have suggested the anti-inflammatory potential of different extracts of *B. oleracea*. A study conducted by Mendoza et al., 35 reported that the methanolic extract of cauliflower exhibited significant anti-inflammatory activity *in vitro*, by inhibiting the production of inflammatory mediators such as nitric oxide, interleukin-1β, and TNF-α. The extracts of broccoli (leaf & stalk) showed anti-inflammatory potential by inhibiting the inhibitory activity against denaturation of albumin 36. The anti-inflammatory and antioxidant potential of ethyl acetate fractions of broccoli florets was reported earlier 14. Similarly, in our study aqueous, acetonitrile and methanol extracts of broccoli exhibited anti-inflammatory activity. Among them, increased anti-inflammatory activity was found in the acetonitrile extracts compared to the methanol and aqueous extracts. Furthermore, boiled broccoli stems showed increased anti-inflammatory activity compared to fresh stems.

Various studies have reported the antimicrobial potential of different *B. oleracea* extracts. Leaf extracts of broccoli showed inhibitory activity against *P. aeroginosa* 37. The extracts of broccoli florets were more potent against *P. aeruginosa* followed by the stem and leaf extracts 38. The antibacterial efficacy of the broccoli was reported against drug resistant *S. aureus* 39. In line with this, we found that aqueous, acetonitrile and methanol extracts of broccoli showed action against *B. subtilis, S. aureus*, and *P. aeruginosa*. Acetone and methanolic extracts of broccoli was more potent against the *B. subtilis* strains 40. Similarly, we also observed that the broccoli extracts were more potent against *B. subtilis,* followed by* S. aureus* suggesting that they were effective against gram positive pathogens.

## Conclusion

Broccoli, a nutritiously rich cruciferous vegetable, is known for its high antioxidant activity and various other bioactivities. We compared the impact of different extracts of cooked and uncooked broccoli samples by evaluating the extraction yield and antioxidant (ABTS, DPPH, and metal ion reduction assays), anti-inflammatory, and antibacterial efficacies. The extraction yields and antioxidant potentials were higher in the acetonitrile and aqueous extracts of broccoli. The anti-inflammatory potential was also found to be higher in the acetonitrile and aqueous extracts of broccoli. TPC and TFC levels were highest in the methanol extract, followed by the aqueous and acetonitrile extracts. All extracts showed potent antibacterial activity against *B. subtilis*, *S. aureus*, and *P. aeruginosa*. Acetonitrile extraction could be the ideal extraction method, as it increases the extraction yield and antioxidant and anti-inflammatory potential. However, detailed time-bound extraction and metabolomic analyses are required to elucidate the bioactive potential of broccoli.

## Supplementary Material

Supplementary figure.

## Abbreviations

TPC: total flavonoid contents
TFC: total flavonoid contents
ROS: reactive oxygen species
ABTS: 2,2'-azino-bis(3-ethylbenzothiazoline-6-sulphonic acid)
DPPH: 2,2-diphenylpicrylhydrazyl
QE: quercetin equivalent
GAE: gallic acid equivalent
