# Supplementary material for: Comprehensive study on the differential extraction and comparison of bioactive health potential of the Broccoli (Brassica oleracea)
Source: Int J Med Sci. 2024 Jan 21;21(4):593–600. doi: 10.7150/ijms.92456 (PMC10920836; doi:10.7150/ijms.92456)
Supplement: Supplementary file 1 — Supplementary figure. [file ijmsv21p0593s1.pdf]

a

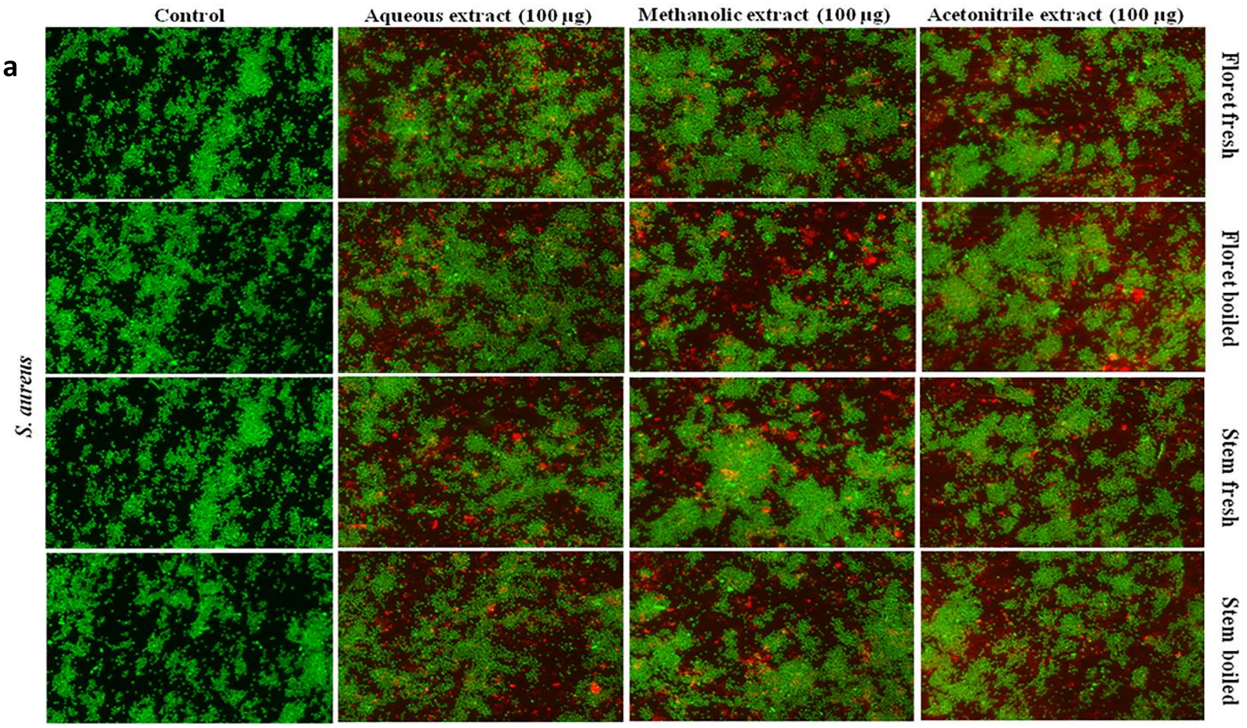
Control
Aqueous extract (100 µg)
Methanolic extract (100 µg)
Acetonitrile extract (100 µg)
Floret fresh
Floret boiled
Stem fresh
Stem boiled
S. aureus

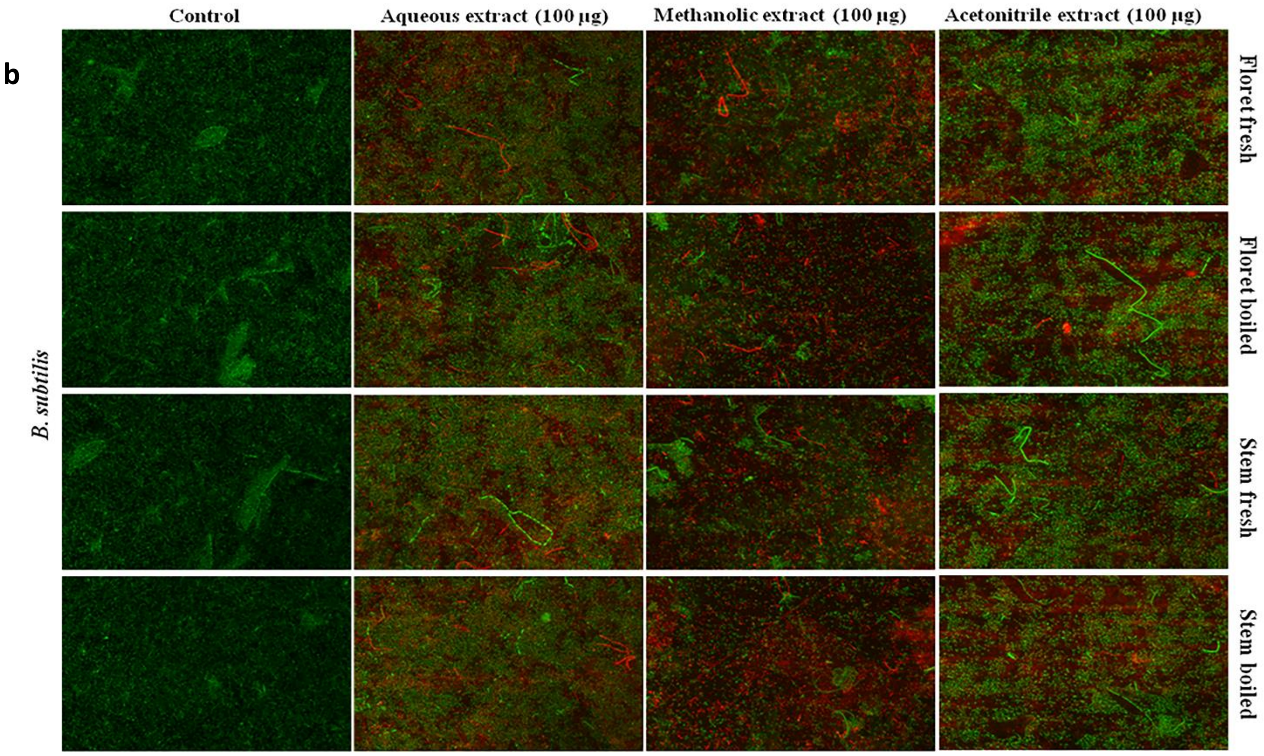
b
Control
Aqueous extract (100 µg)
Methanolic extract (100 µg)
Acetonitrile extract (100 µg)
B. subtilis
Floret fresh
Floret boiled
Stem fresh
Stem boiled

c

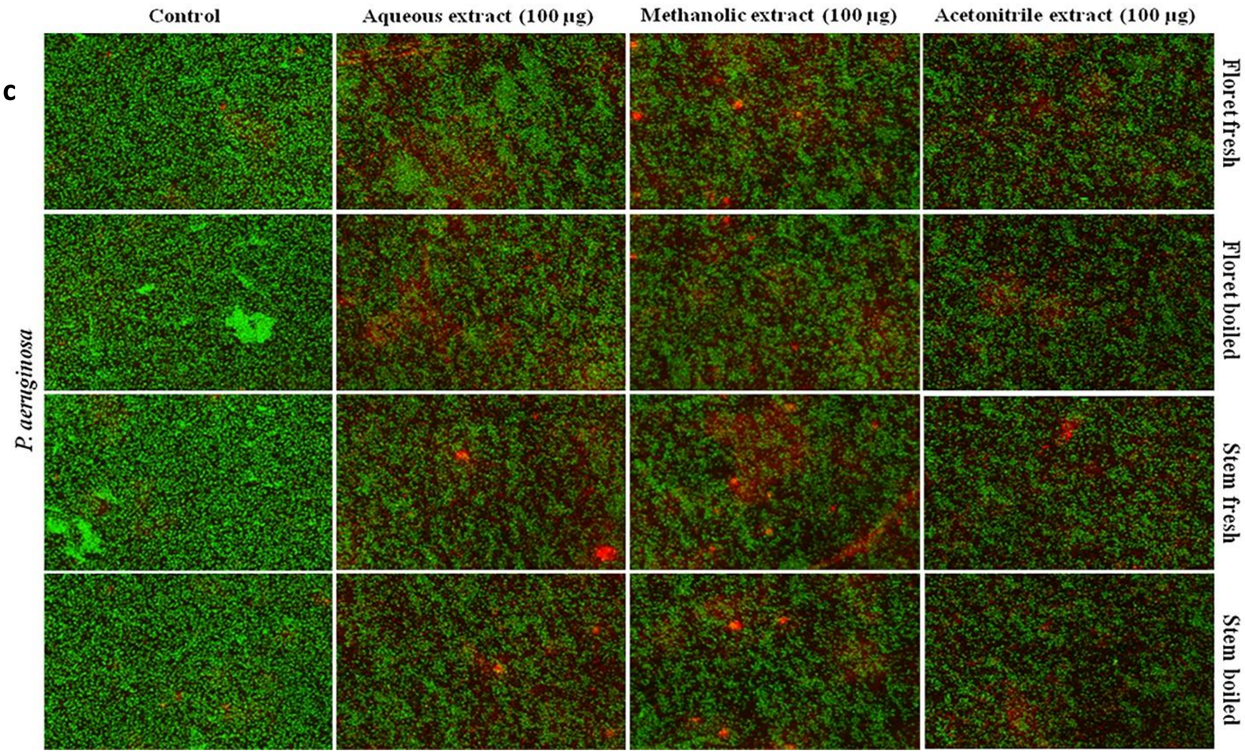
Control
Aqueous extract (100 µg)
Methanolic extract (100 µg)
Acetonitrile extract (100 µg)
Floret fresh
Floret boiled
Stem fresh
Stem boiled
P. aeruginosa

d

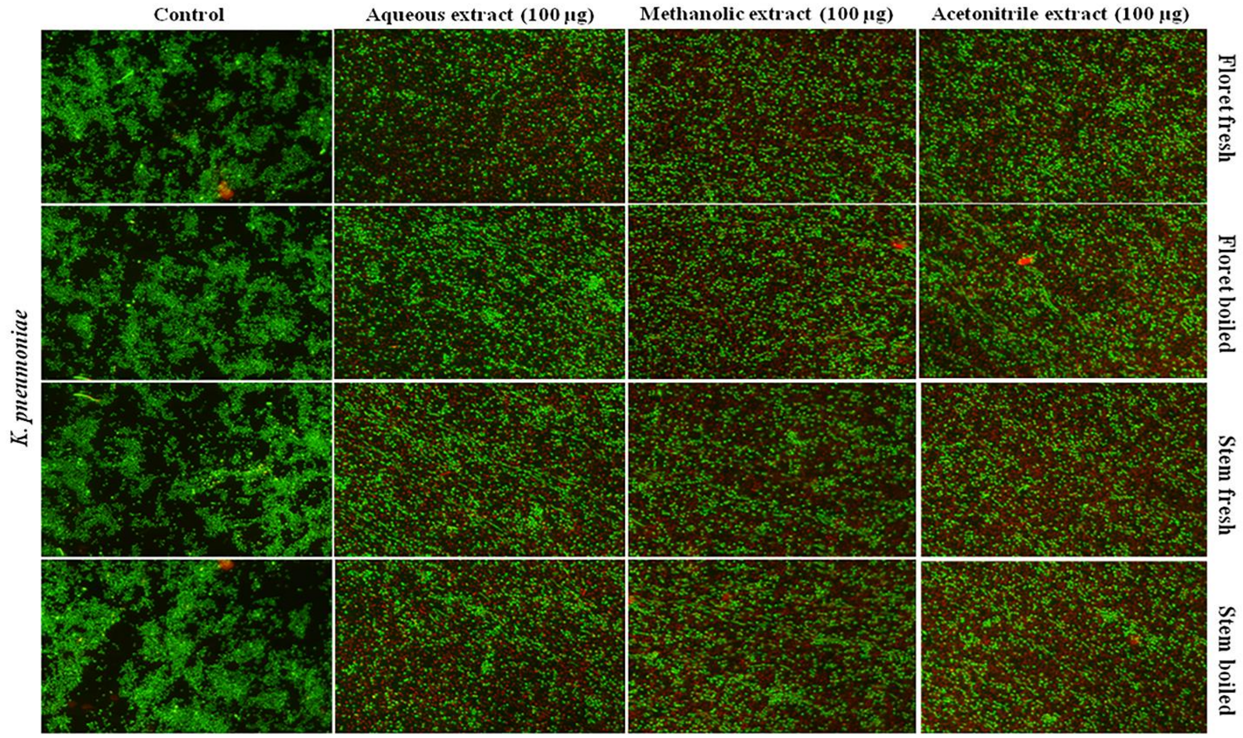

Figure S1. Antibacterial potential of different extracts of broccoli against a. *Staphylococcus aureus* b. *Bacillus subtilis* c. *Pseudomonas aeurginosa* d. *Klebsiella pneumoniae* using live and dead cell assay.
